# Structured reporting in radiology: a systematic review to explore its potential

**DOI:** 10.1007/s00330-021-08327-5

**Published:** 2021-10-15

**Authors:** J. Martijn Nobel, Koos van Geel, Simon G. F. Robben

**Affiliations:** 1 Department of Radiology, Maastricht University Medical Center+, Postbox 5800, 6202 AZ Maastricht, the Netherlands; 2 Department of Educational Development and Research and School of Health Professions Education, Maastricht University, Maastricht, the Netherlands; 3 Department of Medical Imaging of Zuyderland Medical Center, Heerlen, the Netherlands

**Keywords:** Radiology, Reports, Neoplasm staging, Magnetic resonance imaging, Multidetector computed tomography

## Abstract

**Objectives:**

Structured reporting (SR) in radiology reporting is suggested to be a promising tool in clinical practice. In order to implement such an emerging innovation, it is necessary to verify that radiology reporting can benefit from SR. Therefore, the purpose of this systematic review is to explore the level of evidence of structured reporting in radiology. Additionally, this review provides an overview on the current status of SR in radiology.

**Methods:**

A narrative systematic review was conducted, searching PubMed, Embase, and the Cochrane Library using the syntax ‘radiol*’ AND ‘structur*’ AND ‘report*’. Structured reporting was divided in SR level 1, structured layout (use of templates and checklists), and SR level 2, structured content (a drop-down menu, point-and-click or clickable decision trees). Two reviewers screened the search results and included all quantitative experimental studies that discussed SR in radiology. A thematic analysis was performed to appraise the evidence level.

**Results:**

The search resulted in 63 relevant full text articles out of a total of 8561 articles. Thematic analysis resulted in 44 SR level 1 and 19 level 2 reports. Only one paper was scored as highest level of evidence, which concerned a double cohort study with randomized trial design.

**Conclusion:**

The level of evidence for implementing SR in radiology is still low and outcomes should be interpreted with caution.

**Key Points:**

*• Structured reporting is increasingly being used in radiology, especially in abdominal and neuroradiological CT and MRI reports.*

*• SR can be subdivided into structured layout (SR level 1) and structured content (SR level 2), in which the first is defined as being a template in which the reporter has to report; the latter is an IT-based manner in which the content of the radiology report can be inserted and displayed into the report.*

*• Despite the extensive amount of research on the subject of structured reporting, the level of evidence is low.*

## Introduction

The area of radiology is an ever innovating field with new applications, such as speech recognition systems and the introduction of Picture Archiving and Communication System (PACS), leading to digitalization and new possibilities in radiology reporting [1, 2]. The recent introduction of different types of structured reporting (SR) further accelerates initiatives in the field of reporting, and many radiology departments use some sort of SR already [3]. The magnitude of this trend and its promotion by large radiological societies, such as the Radiological Society of North America (RSNA) and the European Society of Radiology (ESR), suggests that this way of reporting is promising and that implementation of SR in clinical practice should be seriously considered [4, 5]. Overall, SR has been thought to be the key to improve clinical and radiological workflow.

The main goal of implementing SR seems to be enhancing the content of the radiological report as well as the reporting process itself. Due to increasing imaging possibilities, larger data sets and the availability of more specific treatments, details become ever more important. The radiological report should arrange this huge amount of information into a readable (legible) text containing the most accurate and specific information that is needed to make accurate decisions to treat the patient best. This renders the radiological reporting process more complicated and time consuming.

To accommodate this increasing demand of information, several tools have been proposed to improve the quality of the radiological report. Standardization tools (RECIST (Response Evaluation Criteria in Solid Tumors), Fleischner glossary, the RADS (Reporting And Data System) collection) [6–8], are created to be more accurate on describing pathology and its extension or evolution, to ensure that the content of the report is accurate. On the other hand, reporting tools, such as structured reporting and reporting guidelines, are constructed in order to enhance the reporting process; this concept is in literature generally referred to as “structured reporting.”

However, before implementation of SR, it is necessary to provide evidence to justify its introduction and implementation in the clinical workflow with a systematic review. As there is a plethora of definitions and interpretations of SR present in literature, a clear definition had to be determined for this review. The definition “structured reporting is an IT-based method to import and arrange the medical content into the radiological report,” as coined by Nobel et al. [9], was used. The main purpose of this systematic review is to explore the level of evidence of structured reporting. Additionally, this review provides an overview on the current status of SR in radiology.

## Materials and methods

A systematic search was conducted according to the Preferred Reporting Items for Systematic reviews and Meta-Analyses (PRISMA) criteria [10], and results were further categorized using a thematic analysis approach [11]. Results were analyzed and interpreted consistently with a textual narrative synthesis to visualize the similarities and differences among various methodologies in study design [12]. The next step was to determine the level of evidence of the studies. Because of the heterogeneity in study design, the simplified grading system (level A/B/C) according to Siwek et al. [13] was used to determine the strength of evidence on which outcomes were based. Randomized controlled trials are considered level A. Level B studies consist of all other evidence except for expert opinions or commentaries, which are level C. The groups were ordered on publication year followed by an alphabetical order. In case of discrepancy, consensus was reached between two authors (J.M.N. and K.G.).

### Literature review protocol

A literature search was conducted by searching PubMed, Embase, and the Cochrane Library up to 10 August 2020. To include relevant papers, a wide search strategy was applied using the combination of the synonyms of ‘radiology’, ‘structure’ and ‘reporting’ (radiol* AND structur* AND report*).

### Eligibility and study selection

All quantitative experimental studies that discussed SR in radiology have been included. After removing duplicates, title and abstract were independently screened on relevance by two authors. The following articles were excluded: articles that did not discuss structured reporting in radiology; comments or expert opinions (level C [13]); articles not in English, German, or Dutch; or those without full text availability. Bibliographies of included studies were searched in order to find additional relevant papers.

### Definition of structured reporting (SR)

The definition “structured reporting is an IT-based method to import and arrange the medical content into the radiological report” [9] was used to frame the field of interest. This definition acknowledges a difference between SR and standardized reporting. Standardized reporting refers to the increase of uniformity of the report content with standardization tools (e.g., RECIST, Fleischner glossary, the RADS collection [6–8]). SR refers to the use of specific tools (structured reporting or reporting guidelines) that can be used to properly build, structure, or fill the radiological report itself. This differentiation is necessary to be able to only include the right studies which change the reporting process and not studies that merely change, for instance, the vocabulary used.

**Fig. 1:**
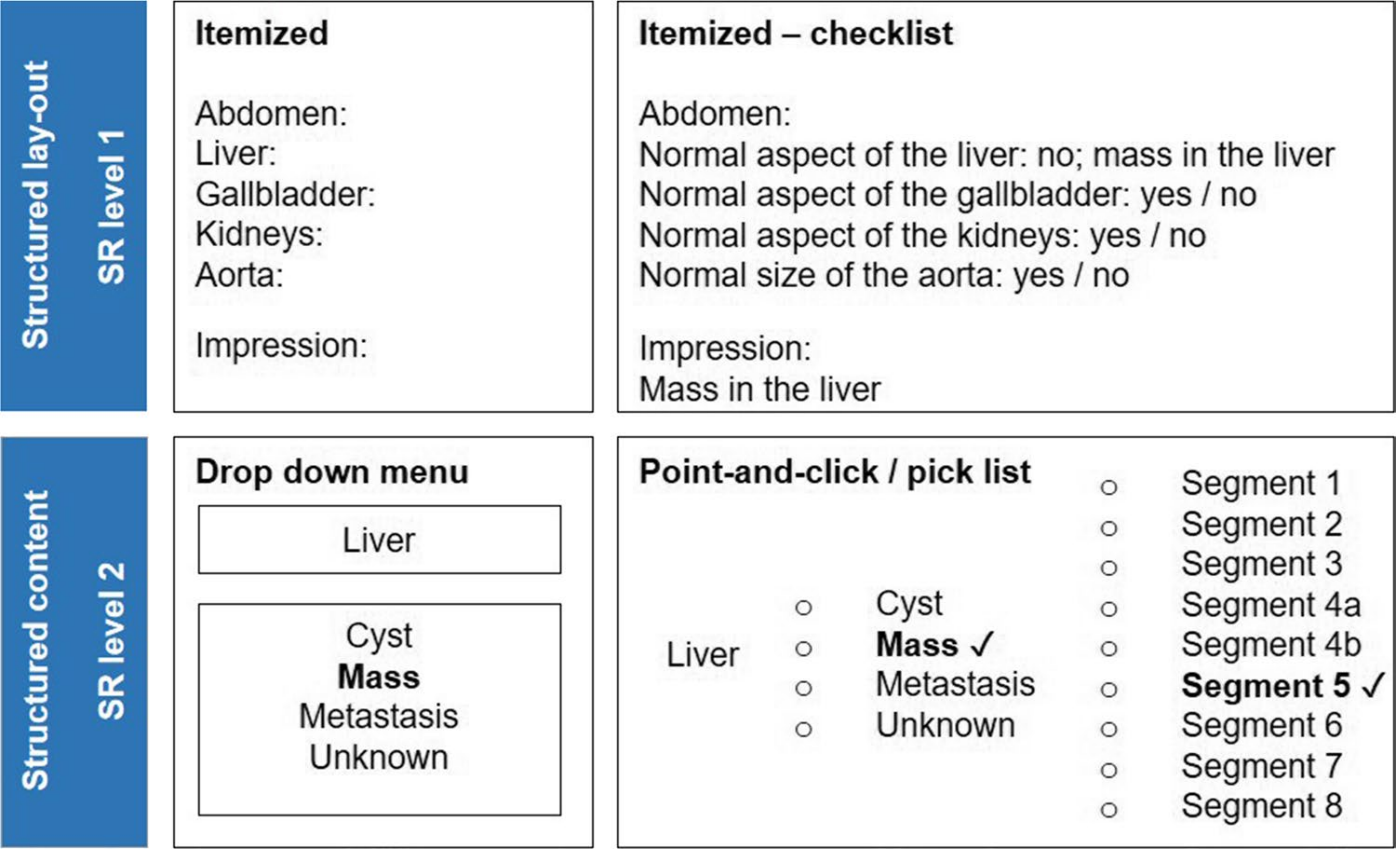
Examples of different levels of structured reporting. SR level 1, structured layout: itemized, itemized-checklist; in these examples, the obligated items or possible options are already stated in the template to ensure its presence. SR level 2, structured content: drop-down menu, point-and-click/pick list; these are examples of IT-based tools to insert specific textual items into the radiological report, for instance with the use of a drop-down menu in which an option can be chosen out of a particular list, or by using a point-and-click/pick list which in turn can open a new point-and-click/pick list option in order to build the report

Additionally, SR is subdivided into structured layout (SR level 1) and structured content (SR level 2) [9]. In this stratification model, structured layout (SR level 1) is defined as being a template or blueprint format in which the reporter has to report or has to adjust to. Structured content (SR level 2) is a manner in which the content of the radiology report can be inserted and displayed into the report (Fig. 1). As such, structured layout (e.g., templates and checklists) and structured content (e.g., drop-down menu, point-and-click or clickable decision trees) highlight the level of IT involvement when implementing SR. This subdivision is used to be able to categorize the types of SR found in the included studies.

## Results

**Fig. 2:**
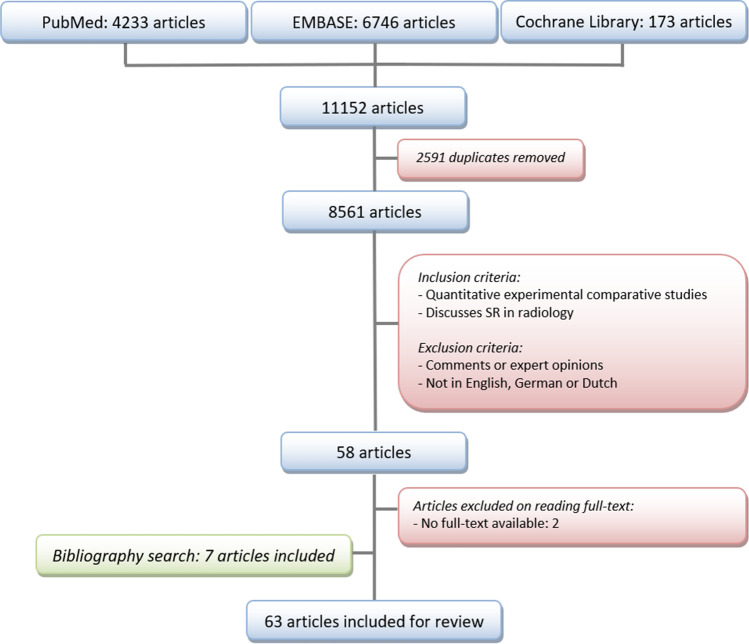
Search flow chart. SR, structured reporting

The literature search retrieved 4233, 6746, and 173 articles (total 11,152) from PubMed, Embase, and the Cochrane Library databases respectively. A total of 2591 duplicates were removed. Title and abstract of 8561 articles were assessed by J.M.N. and K.G., which resulted in 58 relevant articles. Full text was available for 56 articles. Bibliography search resulted in 7 additional studies, leading to a total of 63 studies that were included (Fig. 2 and Table 1). No reviews were found. Due to the heterogeneity of included studies, it was neither possible to perform a meta-analysis nor to pool the results.

**Table 1:** Study characteristics. Overview of articles with level A and B evidence which studied structured reporting in radiology. Presented is the level of evidence, control group, intervention, subspecialty/field, indication, modality and outcome(s)

	Level of evidence	Control	Intervention		Subspecialty/field	Indication	Modality	Outcome(s)
Structured layout (SR level 1) — one template								
Dimarco et al. (2020) [14]	B	Free text		Structured itemized template with four parts and several key items	Abdomen	Pancreatic ductal adenocarcinoma	CT	Significant reduction of missing morphological and vascular features Improvement inter-reader agreement
Gupta et al. (2020) [15]	B	Free text		Added 14 essential parameters	Abdomen	Rectal cancer staging	MRI	Significant report quality improvement Referring provider satisfaction improved
McFarland (2020) [16]	B	Free text		Free-form structured itemized templates	Abdomen	Various	CT	Less reporting errors potentially reducing The report word length did not differ
Olthof et al. (2020)[17]	B	Free text		Additional template with key items for critical findings	Neurology	CNS metastasis	MRI	Automated insertion of context-dependent data and required elements is feasible Guideline adherence concerning critical findings improved
Alessandrino et al. (2019)[18]	B	Free text		Adding key features concerning inherited neuromuscular disorders	Musculoskeletal radiology	Lower limb inherited neuromuscular disorder	MRI	More clinically relevant disease management information
Benson et al. (2019) [19]	B	Free text		Structured template with three options to score CNS metastasis after RT	Neurology	CNS metastasis	MRI	Decreasing non-specific description Improving discrete characterization Usage of non-specific language usage did not differ
Gore et al. (2019)[20]	B	Free text		Template with headings according to BT-RADS	Neurology	Brain tumor (BT-RADS)	MRI	Perception improvement among radiologists and referring providers
Liu et al. (2019) [21]	B	Free text		Structured itemized template with key features and standardized entries	Abdomen	Endometrial cancer	MRI	Increasing radiologists’ work efficiency and gynecologists’ satisfaction
Wetterauer et al. (2019) [22]	B	Free text		Structured reports with PI-RADS key features	Abdomen	Prostate cancer (PI-RADS)	MRI	Urologists’ surgical planning was facilitated by better assessing exact tumor location Improved satisfaction referring physician
Bink et al. (2018)[23]	B	Free text		Itemized template (17 tumor items)	Neurology	Brain tumor staging	MRI	Template ensured reliable detection of all relevant predefined items and reproducible documentation
Griffin et al. (2018) [24]	B	Free text		Itemized template with TI-RADS and/or management integration	Head and Neck	Thyroid nodules (TI-RADS)	Ultrasound	Better feature description ACR TIRADS usage substantially improved management recommendations
Magnetta et al. (2018)[25]	B	Free text		Itemized template using PI-RADS	Abdomen	Prostate (PI-RADS)	MRI	Improved communication and clinical report impact with referring urologists
Olthof et al. (2018) [26]	B	Free text		Itemized RECIST template	Various	RECIST	CT	Combination of optimized workflow, subspecialization and SR led to significantly better report quality
Poullos et al. (2018) [27]	B	Free text		Itemized template	Abdomen	Hepatocellular carcinoma	CT	Assessment of transplant suitability improved using Milan criteria
Tersteeg et al. (2018)[28]	B	Free text		Itemized template with incorporated guidelines and key features	Abdomen	Rectal cancer staging	MRI	More complete report
Flusberg et al. (2017)[29]	B	Free text		Itemized template incorporating including LI-RADS	Abdomen	Hepatocellular carcinoma (LI-RADS)	MRI/CT	More comprehensive and consistent reporting
Franconeri et al. (2017)[30]	B	Free text		Disease-specific itemized template	Abdomen	Uterine fibroid	MRI	Fewer key features were missed More helpful for treatment planning and understanding
Pysarenko et al. (2017)[31]	B	Free text		Template with 8 itemized key-elements	Abdomen	Various	Ultrasound	Improved reimbursement
Wildman-Tobriner et al. (2017) [32]	B	Free text		Itemized template	Abdomen	IBD	CT	Key feature reporting improved Minimal impact on accuracy SR reports were preferred by referring physicians
Wildman-Tobriner et al. (2017)[33]	B	Free text		Itemized template with 15 key elements	Abdomen	Pediatric Crohn’s disease	MRI	Significantly increasing on key features mentioning Referring clinicians subjectively preferred SR
Dickerson et al. (2016)[34]	B	Free text		Itemized template with 12 key features	Brain	MS	MRI	Increased rate relevant findings Standardized reports are preferred by neurologists
Brook et al. (2015) [35]	B	Free text		Itemized template with 12 key features	Abdomen	Pancreatic cancer	CT	Superior evaluation Facilitated surgical planning Increased surgeons confidence concerning tumor resectability
Sahni et al. (2015) [36]	B	Free text		Template with 14 itemized quality measures	Abdomen	Rectal cancer staging	MRI	Report quality improved, 30% of reports remained unsatisfactory
Silveira et al. (2015)[37]	B	Free text		Itemized template and computer-aided diagnosis	Abdomen	Prostate	MRI	Improving report quality Improving contrast enhancement kinetic curve
Lin et al. (2014)[38]	B	Free text		Itemized checklist-based template	Neurology/trauma	Cervical spine	CT	Significant decrease in missed non-fracture findings No change in missed fractures
Marcovici et al. (2014)[39]	B	Free text		Prepopulated itemized checklist template	Thorax	Various	X-ray	Templates are more complete and more effective
Powell et al. (2014) [40]	B	Free text		Itemized checklist-based template	Neurology/trauma	Maxillofacial	CT	No improvement on report accuracy of radiology residents Focused training, checklist flexibility, and an adjustment period are important Only mandatory checklists were readily adopted by residents
Fraser et al. (2013) [41]	B	Free text		Itemized template with different options (paper)	Head and Neck	Cervical lymphadenopathy	Ultrasound	Increased report streamline
Structured layout (SR level 1) — multiple templates								
Chung et al. (2020)[42]	B	Free text		Seven different cross-divisional standardized structured reports	Thorax	Various	X-ray	Improvement of economic gains and projected radiologist time
Hanna et al. (2016) [43]	B	Free text		Seven different itemized templates (4 CTs, 2 X-rays, 1 ultrasound)	Emergency	Various	Various	Decrease of dictation time Decrease of total word length in some cases Mixed impact on total reporting time
Hawkins et al. (2014)[44]	B	Free text		228 different prepopulated templates which may consist a pick list, fill-in-field and/or prose dictation	Various	Various	Various	Carefully constructed structured reports can help reducing errors
Larson et al. (2013)[45]	B	Free text		228 different prepopulated templates which may consist a pick list, fill-in-field and/or prose dictation	Various	Various	Various	High implementation adaptation rate
Hawkins et al. (2012)[46]	B	Free text		Different prepopulated templates	Various	Various	Various	Prepopulated reports alone do not affect error rate or dictation time of radiology reports
Schwartz et al. (2011) [47]	B	Free text		Different itemized templates	Various	Various	CT	Better content and greater clarity for radiologists and referring clinicians
Liu et al. (2003) [48]	B	Free text		Different menu-based templates	Various	Various	Various	Faster report turn-around time Less transcription errors and lower transcription costs
Structured layout (SR level 1) — hypothetical research								
Dabrowiecki et al. (2020) **[**49]	B	Free text		One negative chest X-ray report compared with one out of four templates	Thorax	Chest	X-ray	Template use resulted in better comprehension by the public Unnecessary follow-up was less likely
Camilo et al. (2019) [50]	B	Free text		Four different templates (one free text, two ultrasound and one CT report)	Abdomen	Various	Ultrasound CT	Structured report with final conclusion/comment is preferred by attending and requesting physicians
Heye et al. (2018) [51]	B	Free text		Three different layouts (structured itemized text, tables, images)	Thorax	Chest	CT	The costumer favors structured reporting
Lather et al. (2017) [52]	B	Free text		Structured itemized template	Thorax	Chest	CT	SR is superior
Travis et al. (2014) [53]	B	Free text		Three different layouts with measurement section	Thorax/abdomen	Various oncological	CT	A separate lesion measurement section is preferred over random mentioning
Krupinski et al. (2011) [54]	B	Free text		Itemized and hierarchical template	Abdomen	Renal abnormalities	CT	A “one-size-fits-all” radiology report format does not exist
Grieve et al. (2008) [55]	B	Free text		Four different templates	Abdomen	Negative examination	Ultrasound	Detailed reports and a radiologists’ opinion is preferred by general practitioners
Sistrom et al. (2005) [56]	B	Free text		Itemized structured templates	Abdomen	Renal calcifications	CT	Equally efficient and accurate for transmitting content
Naik et al. (2001) [57]	B	Free text		Three itemized with difference in completeness	Abdomen	Various	Ultrasound	Improved facilitation of complete documentation Itemized reports are preferred by radiologists and referring clinicians
Structured content (SR level 2)								
Johnson et al. (2010) [58]^a^	A	Free text		Point-and-click system used to build a sentence in the structured report	Neurology	Possible stroke	MRI	No improvement in report clarity by attending physicians
Johnson et al. (2009) [59]^a^	A	Free text		Point-and-click system used to build a sentence in the structured report	Neurology	Possible stroke	MRI	Report accuracy and completeness did not improve
Aase et al. (2020) [60]	B	Free text		Template checklist with six pick list options concerning incidental pulmonary nodule description	Thorax	Pulmonary nodule	CT	Increased documentation compliance Better follow-up process Low utilization rates
Alper et al. (2020) [61]	B	Free text		Template with pick list options with preferred terms for abdominal organs normal finding mentioning	Abdomen	Various	CT/MRI	Better use of preferred/acceptable phrases Decreased use of equivocal terms
Kim et al. (2020) [62]	B	Free text		Template-based structured reports with point-and-click menus including standard elements used in a densitometry report	Nuclear radiology	Osteoporosis	DXA	Shorter reporting times Increased report quality
Tuncyurek et al. (2019) [63]	B	Free text		Template with pick list options to describe 12 key features of pelvic MRI for perianal fistulizing disease	Abdomen	Perianal fistulizing disease	MRI	Fewer key features were missed More complete, clear and helpful for treatment planning
Armbruster et al. (2018) [64]	B	Free text		Clickable decision trees that function as a checklist and to use for building automatically semantic sentences	Head and neck	Petrous bone	MRI	Increases completeness and quality Satisfaction of referring physicians improved
Sabel et al. (2018) [65]	B	Free text		Clickable decision trees on several items with several subitems concerning vascular status	Vascular	Lower extremities	CTA	Superior clarity, completeness, clinical relevance, and usefulness rated by referring clinicians
Schoeppe et al. (2018) [66]	B	Free text		Clickable decision trees in which outcomes were used to create semantic sentences and were displayed in the report	Abdomen	Swallowing disorders	Swallowing studies	Increases detailed information and facilitation of information extraction Better assisting clinical decision-making
Schöppe et al. (2018) [67]	B	Free text		Clickable decision trees for specific items concerning (degenerative) osteoarthritis of the glenohumoral joint used to create semantic sentences used in the report	Musculoskeletal radiology	Shoulder	X-ray	May be a useful tool in clinical decision-making
Shaish et al. (2018) [68]	B	Layout template		Drop-down menus which were used as template to describe individual lesion characteristics concerning PI-RADS	Abdomen	Prostate	MRI	PI-RADS adherence improved May increase diagnostic performance
Gassenmaier et al. (2017) [69]	B	Free text		Template with findings and impression section with clickable decision trees with several levels	Musculoskeletal radiology	Shoulder	MRI	Improved readability Improved linguistic quality
Norenberg et al. (2017) [70]	B	Free text		Clickable decision trees used to describe 13 key features	Abdomen	Rectal cancer	MRI	Facilitates surgical planning Higher satisfaction level of referring surgeons about report correctness and clinical decision making
Sabel et al. (2017) [71]	B	Free text		Clickable decision trees containing observations with standardized subheadings in a consistent order	Thorax	Pulmonary embolism	CTA	Superior in clarity, better content and clinical utility
Walter et al. (2015) [72]	B	Free text		Pick list about coronary calcifications added to a structured report with normal and abnormal default standard terminology which auto-populates the report	Cardio	Coronary calcifications	CT	Improved accuracy of coronary calcification mentions
Schweitzer et al. (2014) [73]	B	Free text		Template with 108 obligated items with drop-down menus and free text option. The report contains highlighted parts when stated as abnormal	Forensics	Whole body	CT	Can act as guideline
Karim et al. (2013) [74]	B	Free text		Different IT-based options were used and included standardized point-and-click menus, including anatomy, measures and additional diagnostic findings listed by organ and dedicated pathology in three different sections with a free text option for personal judgment	Vascular	Abdominal aortic aneurysm	CTA	Decrease in average reporting time Ease of use may lead to more accurate decision support
Barbosa et al. (2010) [75]	B	Free text		Pick list reporting system on 8 descriptive items necessary for thyroid nodule characterization	Head and neck	Thyroid	Ultrasound	Information transmission improved for radiologists and referring clinicians
Hasegawa et al. (2010) [76]	B	Free text		Pick list items and particular modifiers for different categories can be entered in templates that link those together	Thorax	Chest	X-ray	Report production time decreased

^a^Identical study population or cohort

*SR*, structured reporting; *SR level 1*, structured layout; *SR level 2*, structured content; *CNS*, central nervous system; *BT-RADS*, Brain Tumor-Reporting And Data System; *PI-RADS*, Prostate Imaging-Reporting And Data System; *TI-RADS*, Thyroid Imaging-Reporting And Data System; *RECIST*, Response Evaluation Criteria in Solid Tumours; *LI-RADS*, Liver Imaging-Reporting And Data System; *RT*, radiotherapy; *IBD*, irritable bowel disease; *MS*, multiple sclerosis

### Thematic data analysis

**Fig. 3:**
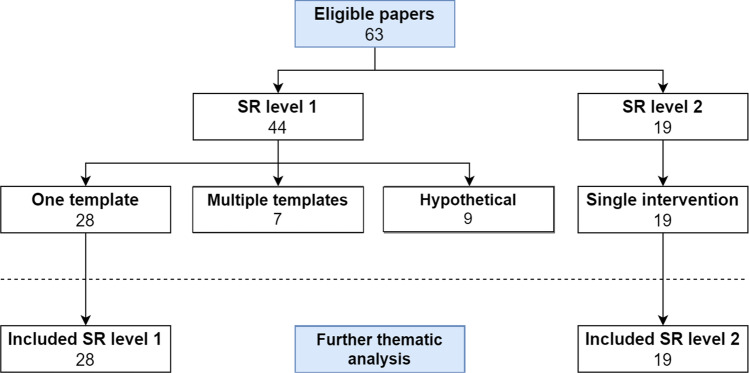
Characteristics of included studies based on SR level. SR level 1, structured layout; SR level 2, structured content

**Fig. 4:**
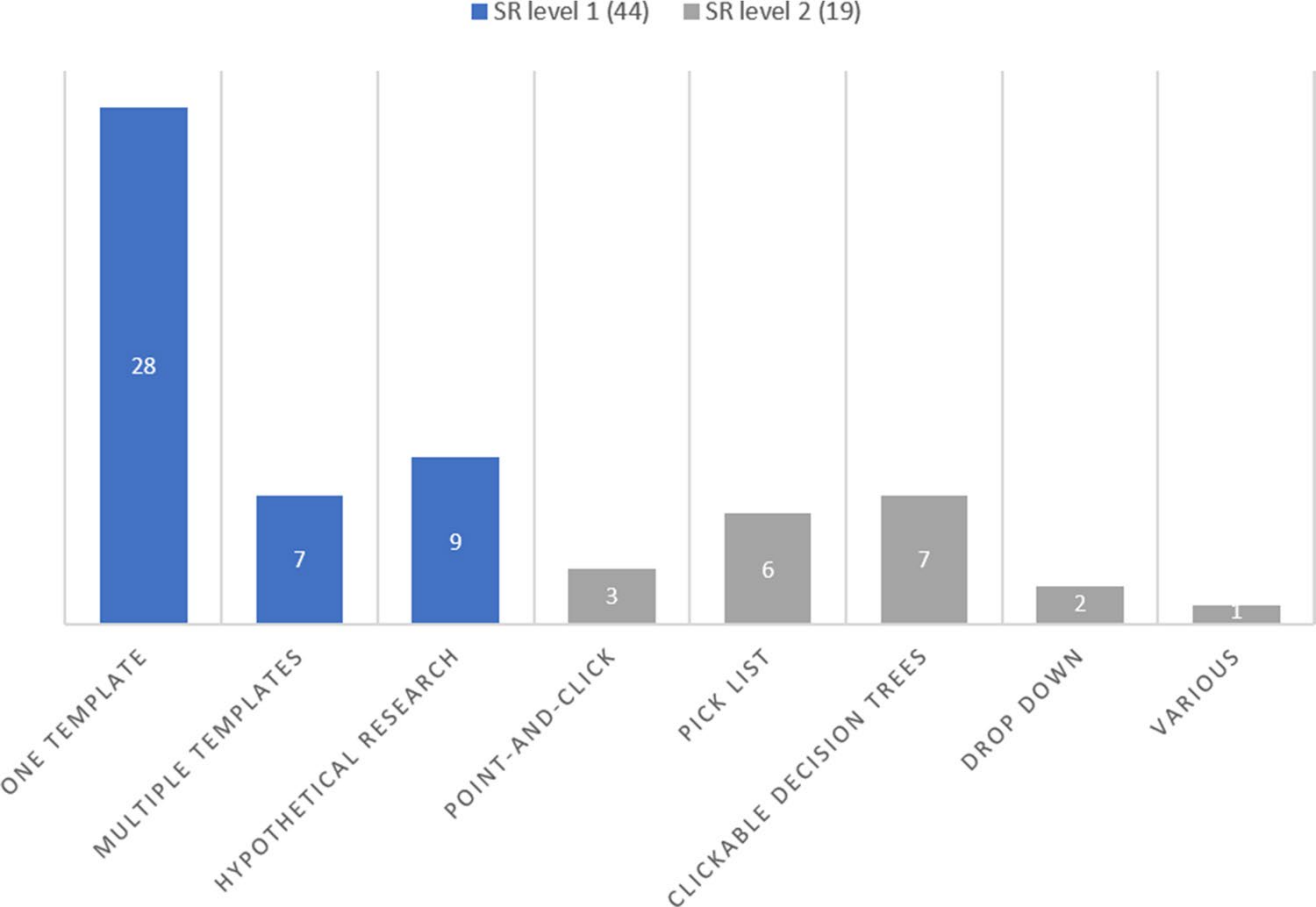
Intervention based on SR level. SR level 1, structured layout; SR level 2, structured content

After inclusion, the 63 studies were grouped into structured layout (SR level 1) and structured content (SR level 2) groups (Fig. 3). Control group, intervention, subspecialty/field, indication, modality, and outcome of each study were assigned. Because of heterogeneity in the structured layout group (SR level 1), this group of 44 studies was subdivided into three subcategories: (1) one template (*n* = 28), (2) multiple templates (*n* = 7), and (3) hypothetical research (*n* = 9) (Table 1, Fig. 3 and Fig. 4).

The first subcategory “one template” consists of studies that implement and compare only one template with a free text report comparison. An example can be an itemized template to report a specific clinical question, such as a magnetic resonance imaging (MRI) for brain tumor staging. The second subcategory “multiple templates” implemented several templates at once in their study before the comparison with free text reports was made. An example can be the implementation of several different templates for different clinical questions, such as implementing templates for computed tomography (CT), ultrasound, and X-ray concerning kidney stones, appendicitis, and heart failure. The third subcategory “hypothetical research” concerned studies that did not actually implement SR in clinical workflow, but assessed clinical or referring preferences on how to present the radiological information in the radiological report.

All 19 structured content (SR level 2) studies were interventional studies using an IT-based method to create the radiological report in the subcategories point-and-click system, pick list, clickable decision trees, drop-down and various (Table 1, Fig. 3 and Fig. 4).

As it is only possible, in an evidence-based manner, to accurately compare one structured reporting tool in one clinical interventional setting at once, only the studies implementing one template from the structured layout group and non-hypothetical studies have been used for further analysis. When not taking into account the hypothetical studies, nor the studies of the multiple template category*,* 28 studies remain on the structured layout level (SR level 1). All 19 structured content (SR level 2) studies were interventional studies using one IT-based method to create the radiological report and were all suitable for further analysis (Table 1, Fig. 3 and Fig. 4). The remaining subcategories (one template SR level 1 and all SR level 2 studies) resulted in 47 studies (Fig. 3).

**Fig. 5:**
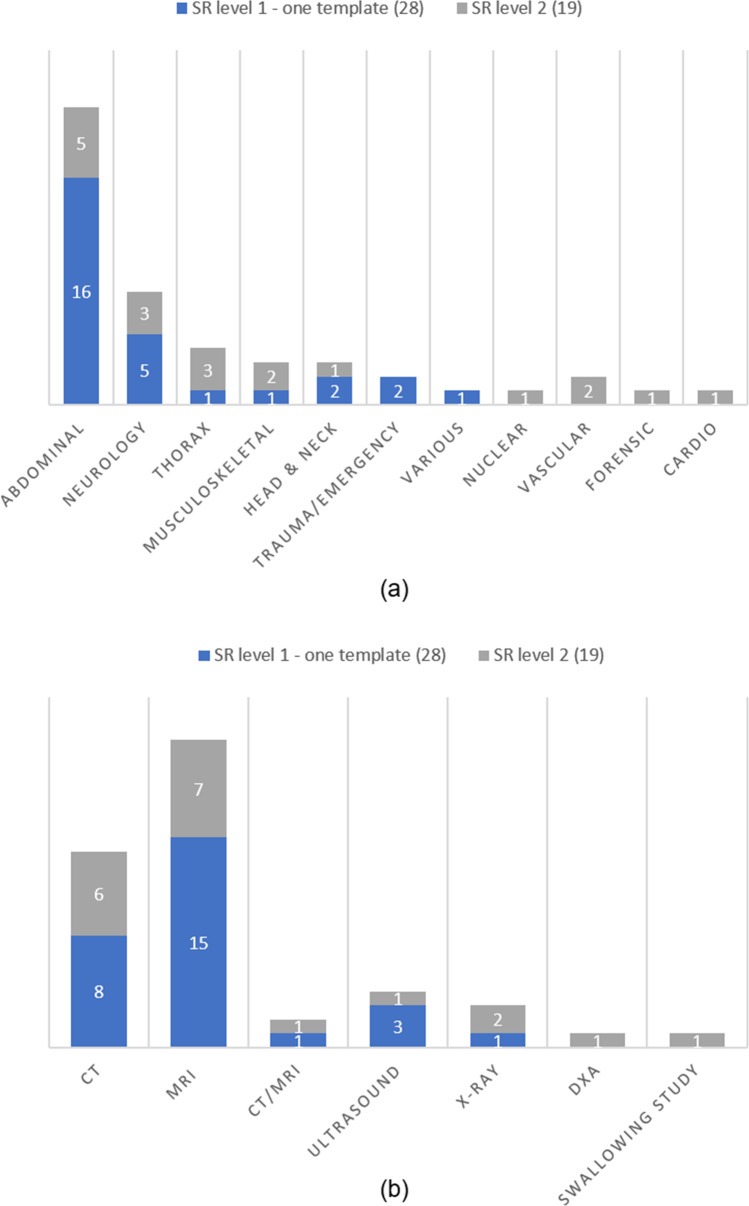
**a** Subspecialty based on SR level and (**b**) modality used based on SR level. All included single intervention studies according to the field of specialty and modality used. SR level 1, structured layout; SR level 2, structured content; DXA, dual-energy X-ray absorptiometry (DXA)

Further analysis of these 47 studies resulted in additional characteristics about subspecialty field and used modalities (Fig. 5a and b). Overall, CT and MRI modalities are mostly used on the subspecialties abdomen and neurology.

### Level of evidence

**Fig. 6:**
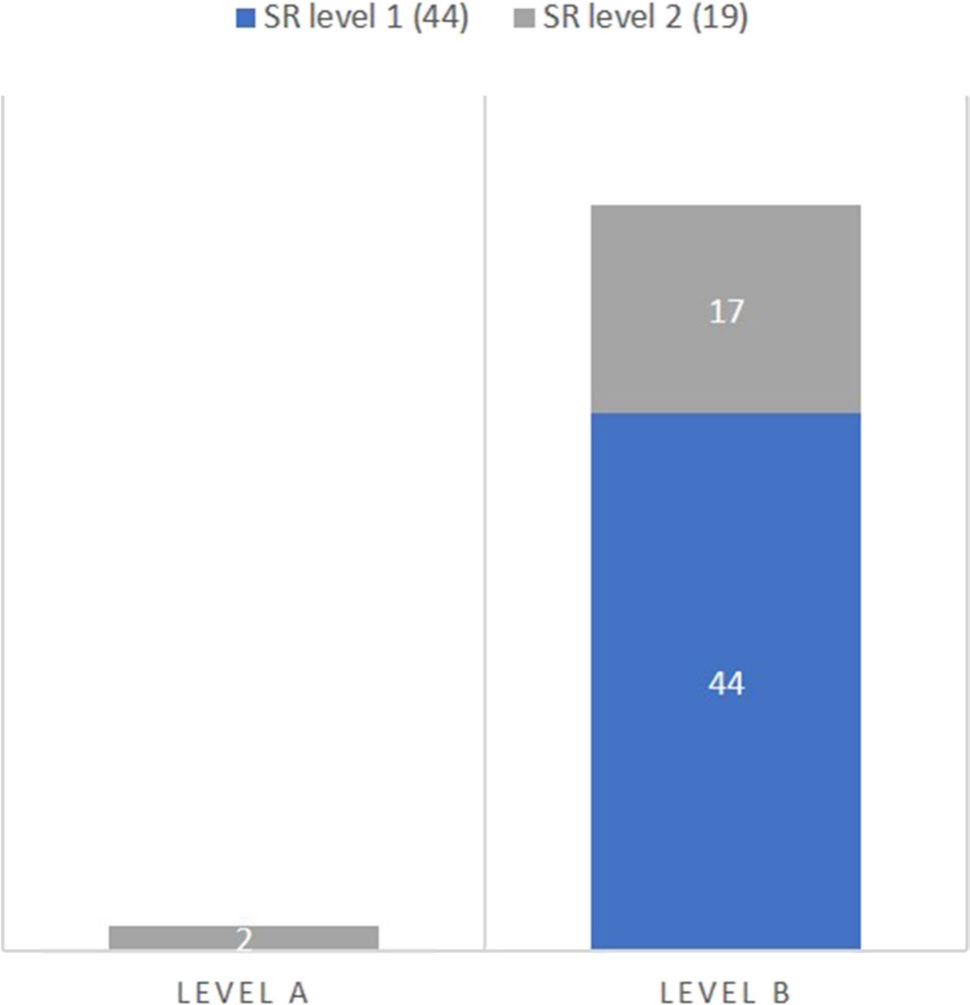
Level of evidence based on SR level. Level A, level A evidence according to Siwek et al. [13]; SR level 1, structured layout; SR level 2, structured content

Two papers (one single study) were scored as level A in the structured content group. All other studies in the structured layout and structured content group were scored as level B evidence (Fig. 6).

### Outcome

The value of outcomes of the studies on structured reporting depends heavily on the level of evidence of these studies. Therefore, the main focus of this study was to determine the level of evidence. However, to create an overview of research done on SR in radiology, main outcomes of included SR studies have been summarized in Table 1.

## Discussion

The main goal of this narrative systematic literature review was to explore the level of evidence of all studies that try to enhance the radiological reporting process by using SR. This also resulted in an overview on the current status of SR in radiology and a summary of its outcomes. To our knowledge, this is the first paper to provide a systematic review of SR in radiology.

### Level of evidence

A double-blinded, randomized controlled trial is considered the highest level of original research (not including systematic reviews or meta-analysis). In our literature search, the only study that approximates this level was the double cohort study with randomized trial design conducted by Johnson et al. [58, 59] and was therefore scored as level A evidence. They compared a point-and-click reporting system (SR level 2) with free text reporting in brain MRI in stroke patients in two papers. This study states that only the way of reporting varied in order to exclude all other interfering factors, thereby only investigating the effect of the change in reporting method. The remaining 61 studies were considered level B evidence, showing an overall low level of evidence.

The hypothetical subcategory studies (*n* = 7) are not implementational but only exploratory of nature. The multiple template studies (*n* = 9) are considered low-level evidence, because it is virtually impossible to confidentially match outcomes to a particular way of reporting, when (a) introducing several templates or reports simultaneously, (b) using different levels of SR, for (c) trying to answer different clinical questions.

However, also the other subcategory studies (one template SR level 1 and all SR level 2 studies), except both level A studies, changed several factors during the implementation of SR, which again can result in some sort of confounding. For instance, many papers describe an expert meeting among radiologists and/or clinicians, or conducted a literature review in order to create a template or pick-list with adequate vocabulary, before implementing SR. This introduced an additional standardizing step next to the implementation of SR in the reporting routine. As a result, both the report content and the reporting manner differed, and outcomes of these studies reflect the effect of the combined interventions. The effects of any individual intervention, however, remain unclear.

Additionally, an expert meeting or literature review before implementing the new reporting manner will likely result in an increase in report quality or accuracy, because the reporter will be guided in stating the correct (newly stated) items necessary for diagnosing when using SR, and thereby enhancing the report content. In this way, confirmation bias can occur, especially when report content quality or accuracy was the main goal of the study, and when outcomes were scored by the same experts that participated in the initial expert meeting.

The aforementioned shows that the study design of the included studies was hampered, resulting in low level of evidence studies. However, despite the fact that most studies are of low evidence, the total amount of published papers show the magnitude of the trend towards structured reporting in radiology.

One of the issues in chosen study design is probably based on the willingness to improve the radiological report as final clinical outcome, rather than searching for the true (single) vehicle that facilitates this.

Furthermore, a reason for the lack of high-level evidence papers can be the fact that proper implementation of SR might be highly case-specific. In radiology, multiple modalities as well as multiple clinical questions coexist and therefore it is possible that a SR tool or a specific SR level is not beneficial for all clinical settings or that it is depending on for instance difficulty level. A point-and-click or clickable decision tree method (SR level 2) may be better for a simple task with only few options, such as describing a thyroid nodule on an ultrasound examination. Likewise, a difficult, extensive clinical question which needs highly specific information or an extensive description, such as the description of a brain tumor on MRI, may suit a template or checklist (SR level 1) better than a point-and-click/pick list. In combination with several vendor-dependent structuring methods on different SR levels, this makes it difficult to choose a specific topic to set up a well-designed study. Also the fact that there are no studies found that compare two different SR methods, but only comparing free text with some sort of SR, shows that research on SR in radiological reporting is still at an exploratory level.

### Current standing and future perspectives

Looking at the levels of SR, in total, 28 studies were performed at the level of structured layout implementing one template and 19 on the structured content level implementing a more IT-based type of SR, which shows that both SR level 1 and 2 are used in clinical studies. It is interesting to see that both levels are being investigated, because it is important to realize that in most cases it is easier, due to its lower IT-demand, to implement a template (SR level 1) in the reporting process than, for instance, implement a drop-down-menu-based report (SR level 2).

When looking at modality and subspecialty, most efforts are made with reports of CT and MRI examinations in the field of abdominal radiology and neuroradiology. An explanation might be the fact that the most important (staging) procedures use CT and MRI as a modality. Perhaps, the abdominal and neuroradiology fields are more suitable for using templates or it can be triggered by the fact that good classification systems or standardization systems already exist in these fields. If this is the case, this highlights the fact that SR is used for standardization by making sure that specific items or classification systems are described or used.

Table 1 shows that SR level 1 (templates) are mainly used to describe key features necessary to stage a particular disease or tumor with a predefined sentence with or without a particular standardization tool. Used standardization tools or classification systems can be found in Table 1, and examples are for instance PI-RADS, LI-RADS and RECIST, but also key elements concerning Crohn’s disease, rectal cancer staging, multiple sclerosis (MS), trauma or head and neck lymphadenopathy are used. Hence, also SR level 2 studies use key feature description or standardization tools (e.g., PI-RADS) to describe specific disease or tumors, such as stroke, pulmonary nodules, rectal cancer, thyroid nodules, or prostatic cancer (Table 1). However, SR level 2 studies use an IT-based system that supports constructing (semantic) sentences, according to the chosen option from the drop-down menu or point-and-click system, in which standardization is almost automatically linked to structured reporting.

When looking at the study outcomes in Table 1, the main goals, incentives, used SR method, and outcomes of each study vary widely, and therefore, pooling of outcomes is difficult. Despite this heterogeneity, this table of outcomes provides a panoramic overview of the present status of SR in radiology.

It shows that most of the included papers show an improvement in outcome when implementing SR. However, when looking at the evidence level, the only level A study [58, 59] did not improve the report clarity, accuracy, and completeness of the report using their point-and-click method. This is an interesting finding and can show that this particular point-and-click system was not beneficial in radiological reporting in this specific setting and concerning this specific outcome. However, the outcome of this study alone is insufficient to state that SR level 2 is not beneficial in radiology reporting, because outcomes seem to be highly case-specific. However, it is also hard to state that SR is beneficial in reporting in radiology when looking at the low level of evidence of all other included studies.

Overall, the level of evidence for SR is low and especially the link between structured reporting and standardization and its different effects on the radiological report is currently overlooked, but is of utmost importance. It seems that improving radiology reporting is more than just implementing SR and that standardization is necessary next to SR, and that both are highly entangled when implementing SR. This is likely caused by the fact that SR is based on a rather strict format in which several (mandatory) items or key features should be reported. Perhaps the question should be whether SR is not just a means to facilitate standardization, rather than that SR is improving the radiological report itself.

As such, high-quality research is necessary to separately investigate the value of all individual factors that are involved in standardization and SR to determine the best type of SR for a specific clinical problem. Investigating the effect of standardization should be prioritized, because it may make sense that improving the content of the report, hence making a complete report with all items referring clinicians are asking for, will likely improve reporting quality. Then, the next question should be how this standardized information should be placed in the radiological report and how we can assure it is inserted correctly. For instance, this can be done with a simple template or checklist (SR level 1), or with a more sophisticated point-and-click system (SR level 2). Finally, it is important to know whether the efforts are beneficial for the patient (e.g., better staging), the referring clinician (e.g., reduced reading time), the reporter (e.g., faster reporting), or for all. Nevertheless, it is possible that this supposed reporting improvement is mainly caused by standardization rather than SR.

### Limitations

First of all, it was difficult to find all relevant implementational studies published on the subject of SR due to ambiguous use of the terms “standardized reporting” and “structured reporting.” To be as complete as possible, as well as to answer the research question best, a prior set definition for SR and its categorization system was used. In addition, a bibliography search was used to search for missed studies after conducting the main search. Because of heterogeneity of the included studies, it was hard to pool the data on a more specific level and therefore a thematic analysis was used. The outcome analysis performed in this paper was limited by the large heterogeneity of outcomes and study design. A more thorough analysis should be done to explore outcome measurements better and to see who (the referring clinician, radiologist or patient) will benefit from SR most, as well as which specific efforts resulted in this outcome.

## Conclusion

Structured reporting is thought to have great potential to improve reporting in radiology. However, due to difficulties in study design there is a lack of high-quality research on this topic resulting in low overall evidence. Future research is needed to explore the individual effects of standardization and SR, as it is questionable whether SR is the solution for improving reporting in radiology or only a means in facilitating standardization.

## Abbreviations

ESR: European Society of Radiology
PACS: Picture Archiving and Communication System
PRISMA: Preferred Reporting Items for Systematic reviews and Meta-Analyses
RSNA: Radiological Society of North America
SR: Structured reporting

## Declarations

### Guarantor

The scientific guarantor of this publication is Simon G.F. Robben, MD, PhD.

### Statistics and biometry

No complex statistical methods were necessary for this paper.

### Methodology

• Systemic literature review
